# Hierarchical Nanogold Labels to Improve the Sensitivity of Lateral Flow Immunoassay

**DOI:** 10.1007/s40820-017-0180-2

**Published:** 2017-12-13

**Authors:** Kseniya Serebrennikova, Jeanne Samsonova, Alexander Osipov

**Affiliations:** 1 Chemistry Faculty, Lomonosov Moscow State University, Leninskiye Gory, Moscow, Russia 119991; 2 National University of Science and Technology “MISiS”, Leninskiy Prospect 4, Moscow, Russia 119049

**Keywords:** Lateral flow immunoassay, Gold nanosphere, Gold nanopopcorn, Gold nanostar, Silver enhancement, Procalcitonin

## Abstract

Lateral flow immunoassay (LFIA) is a widely used express method and offers advantages such as a short analysis time, simplicity of testing and result evaluation. However, an LFIA based on gold nanospheres lacks the desired sensitivity, thereby limiting its wide applications. In this study, spherical nanogold labels along with new types of nanogold labels such as gold nanopopcorns and nanostars were prepared, characterized, and applied for LFIA of model protein antigen procalcitonin. It was found that the label with a structure close to spherical provided more uniform distribution of specific antibodies on its surface, indicative of its suitability for this type of analysis. LFIA using gold nanopopcorns as a label allowed procalcitonin detection over a linear range of 0.5–10 ng mL^−1^ with the limit of detection of 0.1 ng mL^−1^, which was fivefold higher than the sensitivity of the assay with gold nanospheres. Another approach to improve the sensitivity of the assay included the silver enhancement method, which was used to compare the amplification of LFIA for procalcitonin detection. The sensitivity of procalcitonin determination by this method was 10 times better the sensitivity of the conventional LFIA with gold nanosphere as a label. The proposed approach of LFIA based on gold nanopopcorns improved the detection sensitivity without additional steps and prevented the increased consumption of specific reagents (antibodies).
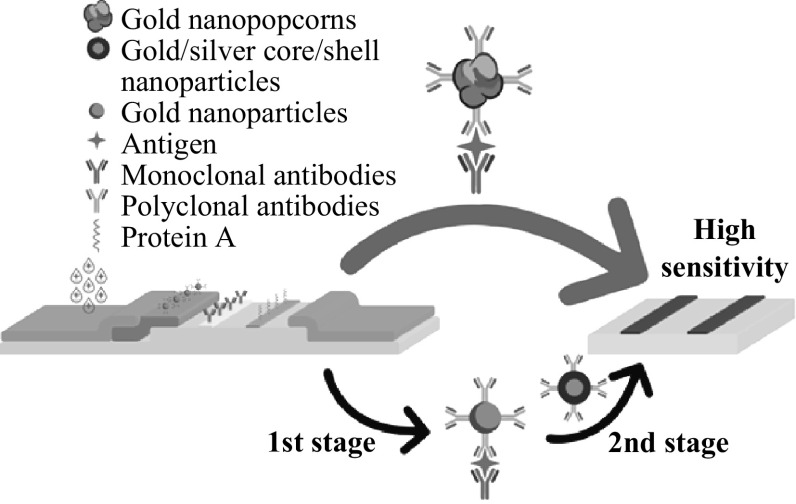

## Highlights

- New types of nanogold labels were evaluated for their improved sensitivity in procalcitonin lateral flow immunoassay (LFIA).
- Gold nanostars and nanopopcorns were applied as a label in a sandwich-format LFIA.
- The use of gold nanopopcorns as a label demonstrated a fivefold increase in sensitivity compared to that associated with the conventional LFIA based on 20-nm gold nanospheres.

## Introduction

The growing interest in the rapid detection of various biologically active substances has necessitated the need for the development of quick methods of analysis. Lateral flow immunoassay (LFIA), one of the most common express methods of analysis, offers advantages such as a short analysis time, long-term stability of test strips, ease of use, and cost-effectiveness, resulting in the expansion of its applications in different fields. Today, LFIA tests are widely used in various sectors, including food, medicine, veterinary, and environment [1, 2]. LFIA is performed on membrane-based strips with pre-immobilized immunoreagents that are activated upon the flow of liquid samples. Despite the advantages of LFIA, some of the rapid tests lack the desired sensitivity and quantitative accuracy. A few approaches have been used to improve LFIA detection sensitivity such as the development of new labels with an easily detectable signal and the use of suitable readout techniques [3, 4].

Studies have reported LFIA labels such as colloidal gold nanoparticles and their modifications, liposomes, latex particles, and quantum dots [5–9]. Of these, colloidal gold nanospheres (GNSs) are widely used [10]. GNSs are inexpensive, owing to the simplicity of their synthesis method, and possess an intense color that may be detected with naked eyes on a test strip. As a rule, larger gold nanoparticles increase the sensitivity of LFIA [11, 12]. Usually, the size of GNSs used in LFIA is less than 50 nm, as larger particles are unstable and require higher antibody concentration to produce labeled reagent [13, 14]. Recent studies have described hierarchical structures of gold nanoparticles with rough surface [15, 16]. Unlike large GNSs, gold nanoparticles such as gold nanorods [17], nano-urchins [18], gold nanopopcorns (GNPNs) and gold nanostars (GNSTs) [19] are more stable because of their complex three-dimensional structures. Moreover, the spherical structure of the nanopopcorn with the large surface area improves the yield of immobilized antibodies on its surface [17]. LFIA based on hierarchical gold nanoparticles are suggested to provide improved sensitivity in the assay. The conjugation of GNSTs and GNPNs with biomolecules requires biocompatible surfactant-free synthesis in aqueous media. Some groups have published surfactant-free method to produce high-yield monodisperse GNSTs and GNPNs [20, 21].

To improve LFIA sensitivity, many efforts were reported, including gold nanoparticles loaded with enzymes [22], a dual gold nanoparticle–antibody conjugate [23], enlargement of immunogold particles [24], and triple lines gold nanoparticle-based LFIA [25]. With these approaches, the limit of detection may be increased up to 1000-fold. In addition, an effective and simple way to amplify LFIA signal based on gold nanoparticles is the silver enhancement method. This method involves silver deposition on gold nanoparticles by combining a silver salt with a reducing agent. Silver enhancement method is widely used in biosensing [26]. The formation of the silver layer on the surface of gold nanoparticles results in the change of the color from red to black and has led to the application of this method in LFIA [27, 28].

Herein, we applied two approaches to improve the sensitivity of LFIA of model protein antigen procalcitonin (PCT). PCT is a highly specific marker used for the diagnosis of bacterial infections and sepsis. Monitoring of PCT levels may allow tailoring of antibiotic therapy in terms of duration and necessity as per the requirements of individual patients. In this study, different types of gold nanoparticles such as GNSs, GNPNs, and GNSTs were prepared and used as labels in LFIA. Moreover, the silver enhancement method was applied to improve the sensitivity of LFIA.

## Experimental Section

### Reagents

Chloroauric acid (HAuCl_4_), sodium citrate, hydroquinone, ascorbic acid, bovine serum albumin (BSA), Tween 20, silver nitrate, and silver enhancer kit were purchased from Sigma-Aldrich (St. Louis, MO, USA). All membranes used for the assembly of the LFIA strip were obtained from MDI (India) and included the sample pad (GFB-R4), conjugate pad (PT-R5), analytical membrane (CNPC-SS12 15 μm), and absorbent pad (AP045). Recombinant PCT, monoclonal anti-calcitonin antibodies, and polyclonal anti-PCT antibodies were purchased from BIALEXA (Moscow, Russia). All salts for the preparation of buffer solutions were obtained from Helicon (Moscow, Russia).

### Synthesis of GNSs, GNPNs, and GNSTs

Gold nanospheres with a size of 20 and 35 nm were obtained by citrate reduction method [29]. After dissolving HAuCl_4_ in water, the solution was stirred while a calculated amount of sodium citrate was added. The solution was boiled for 15 min and then cooled to room temperature in the dark.

Larger GNSs and GNPNs were prepared with the seeding approach [21, 30, 31]. For the synthesis of gold seeds, 2.7 mL 1% sodium citrate solution was added to 100 mL boiling solution of 0.01% HAuCl_4_ and stirred for 15 min. To prepare large GNSs, an appropriate volume of gold seeds was added to the solution of 0.01% HAuCl_4_ under constant stirring, followed by the quick addition of 22 μL 1% sodium citrate and 100 μL 0.03 M hydroquinone. The mixture was stirred for 15–30 min. For the synthesis of GNPNs, 0.5 mL gold seeds, 220 μL 1% sodium citrate, and 1 mL 0.03 M hydroquinone were added dropwise to 0.01% HAuCl_4_ solution. The mixed solution quickly turned blue and was stirred for 30 min.

We prepared GNSTs with the seed-mediated growth method described by Yuan et al. [20]. The seed solution was obtained by adding 15 mL 1% sodium citrate to 100 mL boiling 1 mM HAuCl_4_ solution under vigorous stirring. In 15 min, the solution was cooled and stored at 4 °C for a long term. To prepare GNSTs, 100 µL seed solution was added to 10 mL 0.25 mM HAuCl_4_ supplied with 10 µL 1 M HCl hydrochloric acid under constant stirring, followed by a quick addition of 100 µL 1 mM silver nitrate and 50 µL 1 M ascorbic acid. The solution was stirred for 30 s as its color rapidly changed from light red to blue.

The size distribution and morphologies of the obtained GNSs, GNPNs, and GNSTs were characterized by transmission electron microscopy (TEM, JEOL JEM-2100, Tokyo, Japan). The digital images were analyzed by Image Tool software (University of Texas Health Science Center at San Antonio, USA).

### Preparation of Gold Nanoparticle–Antibody Conjugates

Polyclonal anti-PCT antibodies (pAb) were used to functionalize gold nanoparticles. The optimal concentrations of antibodies were chosen according to flocculation curves obtained for each type of gold nanoparticle. For the preparation of anti-PCT pAb labeled with GNSs (pAb-GNS), GNPNs (pAb-GNPN), and GNSTs (pAb-GNST), 1 mL of anti-PCT pAb at optimal concentration (10–40 µg mL^−1^) was added dropwise to 10 mL colloidal gold solution at pH 7.5. The resulting mixture was incubated for 30 min at room temperature and treated with 0.2% BSA (final concentration) for 30 min under constant stirring. The resulting conjugates (pAb-GNS, pAb-GNPN, and pAb-GNST) were concentrated by centrifugation, resuspended in 10 mM phosphate-buffered saline (PBS), and supplied with 0.1% BSA, 10% sucrose, and 0.01% sodium azide. All probes were stored at 4 °C until further use.

### Assembling of LFIA Test Strips

**Fig. 1:**
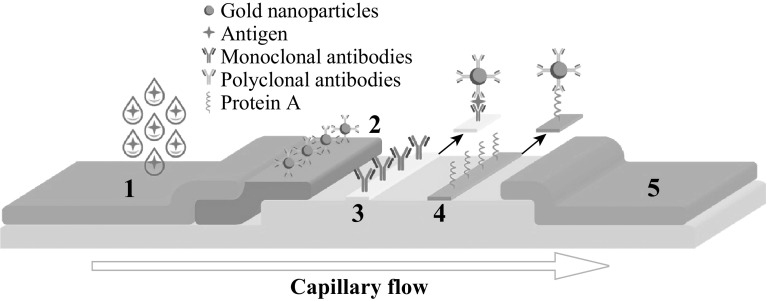
Scheme of LFIA using gold nanoparticles as labels: (1) Sample pad, (2) Conjugate pad, (3) Test line, (4) Control line, (5) Absorbent pad

We assembled LFIA strips (75 × 4 mm) with absorbent pad, conjugate pad, analytical membrane, and sample pad (Fig. 1). The test line on the analytical nitrocellulose membrane was formed by spreading the solution of specific monoclonal antibody (mAb) in PBS by programmable automatic BioJet dispenser (BioDot Inc., USA). Protein A (0.5 mg mL^−1^) was dispensed at 5 mm distance from the test line to form a control line. The strips were dried for 24 h at room temperature.

### LFIA Procedure

Procalcitonin standard solutions at 0.1, 0.5, 1, 2, 5, 10, and 50 ng mL^−1^ concentrations were prepared from 1 mg mL^−1^ PCT stock solution in PBS containing 0.05% Tween 20. A test strip was placed on a horizontal surface, and 120 µL PCT standard solution was added on the sample pad to perform LFIA. All standard solutions were analyzed in duplicates. The intensities of colored test and control lines of the strip were detected in 10 min. Test strips were scanned and obtained line intensities were digitized by Scion Image software.

### Silver-Enhanced LFIA Procedure

The procedure of silver-enhanced LFIA based on 20 nm GNSs was performed with silver enhancer kit according to the manufacturer’s instruction. Briefly, silver nitrate and hydroquinone solutions were mixed in a ratio 1:1 immediately before use and applied to the analytical membrane. After 5–10 min, the test strip was rinsed with distilled water, fixed for 2–3 min in sodium thiosulfate solution, and rinsed again. In 10 min, the test strips were scanned to analyze the signal intensities.

## Results and Discussion

In this study, PCT was used as a model protein antigen for the comparative study of LFIA with nanospheres and hierarchical structures of nanogold labels (nanopopcorns and nanostars). PCT is an important biomarker that exhibits greater specificity than other pro-inflammatory markers (e.g., cytokines) in identifying patients with sepsis and may be used in the diagnosis of bacterial infections. A serum PCT level higher than 10 ng mL^−1^ may be indicative of septic shock. PCT concentrations in sepsis reach values between 2 and 10 ng mL^−1^ PCT values between 0.15 and 2 ng mL^−1^ do not exclude an infection because localized infections (without systemic signs) may be associated with such low levels.

To detect PCT by LFIA, the sandwich scheme of analysis was applied (Fig. 1). The specific antibodies were immobilized in the test zone of the analytical membrane, and the conjugate pad was impregnated with the paired detection antibodies labeled with gold nanoparticles. The application of the solution containing the antigen to the test strip (sample pad) resulted in the binding of paired antibodies labeled with gold nanoparticles to PCT in the sample to form labeled antigen–antibody complex. This complex moved by capillary forces through the area containing the test zone, wherein the labeled complex attached to the immobilized specific antibodies and formed a sandwich complex. This sandwich complex may be observed as a colored band, and the color intensity of the band was directly proportional to PCT concentration in the samples.

In this study, seven conjugates, namely antibodies labeled with GNPNs, GNSTs, and GNSs of different sizes were prepared and used in LFIA for PCT detection. To compare the effect of different labels on the assay sensitivity, LFIAs based on different gold nanoparticles and silver enhancement procedure were performed.

### Characterization of GNSs, GNPNs, and GNSTs

**Fig. 2:**
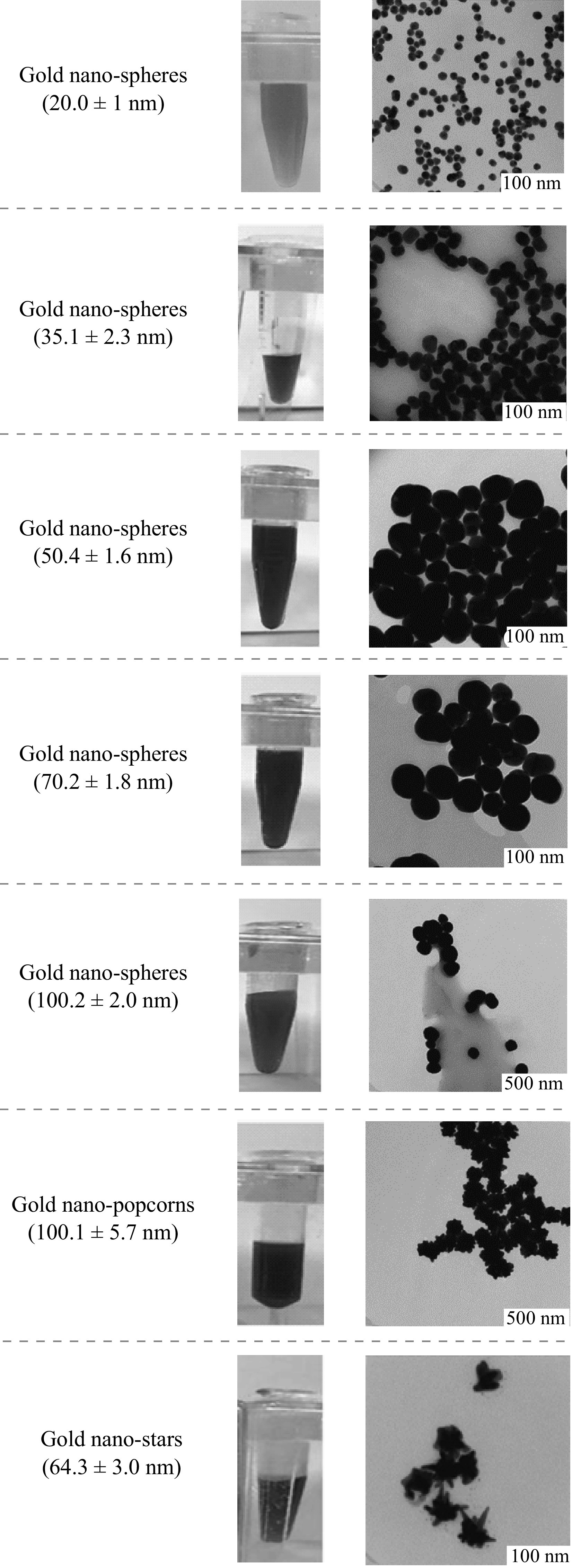
Characterization of GNSs, GNPNs, and GNSTs: photograph of colloidal solutions and TEM images of gold nanoparticles

**Fig. 3:**
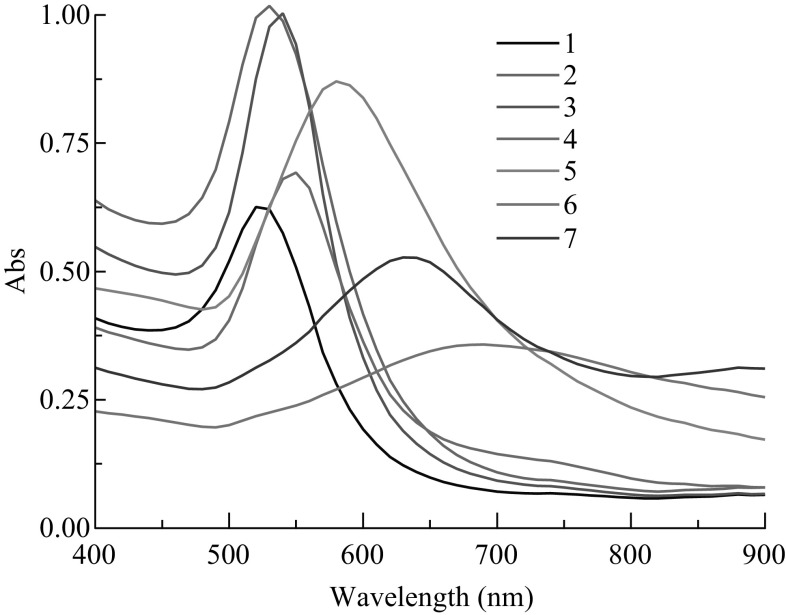
UV–Vis spectra of gold nanoparticles: (1) GNSs 20.0 ± 1 nm, (2) GNSs 35.1 ± 2.3 nm, (3) GNSs 50.4 ± 1.6 nm, (4) GNSs 70.2 ± 1.8 nm, (5) GNSs 100.2 ± 2.0 nm, (6) GNPNs 100.1 ± 5.7 nm and (7) GNSTs 64.3 ± 3.0 nm

The color of gold nanoparticles strongly depends on their sizes and shapes. Figure 2 displays images of colloidal solutions and TEM. The calculated average diameters of GNSs were 20.0 ± 1, 35.1 ± 2.3, 50.4 ± 1.6, 70.2 ± 1.8, and 100.2 ± 2.0 nm, while those for GNPNs and GNSTs were 100.1 ± 5.7 and 64.3 ± 3.0 nm, respectively. UV–Vis spectra demonstrated maximal absorbance at 520 (20 nm), 530 (35 nm), 537 (50 nm), 547.5 (70 nm), and 582.7 nm (100 nm) wavelengths for GNSs and 683 and 633 nm for GNPNs and GNSTs (Fig. 3), respectively. All the obtained samples exhibited good colloidal stability and homogeneity in terms of composition and size.

### Optimization of LFIA Conditions

**Fig. 4:**
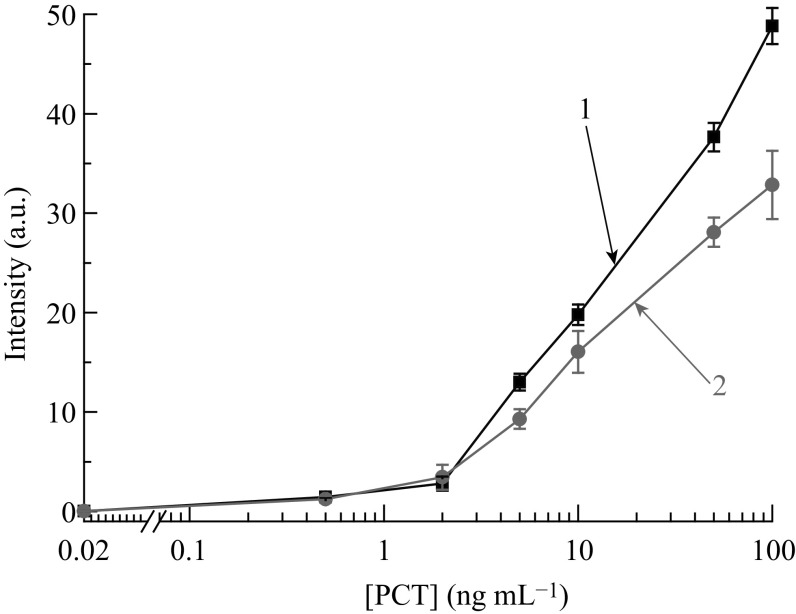
Calibration curves of PCT LFIA for different combinations of antibodies: (1) immobilized antibodies–mAb, detection antibodies–pAb, and (2) immobilized antibodies–pAb, detection antibodies–mAb

All gold nanoparticles (GNSs, GNPNs, and GNSTs) were used as labels in LFIA, and the resulting antibody-nanoparticle conjugates were impregnated in the conjugate pad. Optimization of the assay system included the choice of specific antibodies, antibody-gold nanoparticle conjugate, and concentration of immunoreagents in the test and control lines. The combination of immunoreagents and their concentrations were chosen based on low background and minimum PCT detection limit. To choose a pair of antibodies that meets the requirements of LFIA, a few clones of mAb and pAb were used and the calibration curves for all combinations were obtained for LFIA with 20 nm GNSs (data not shown). The highest assay sensitivity with a PCT detection limit of 2 ng mL^−1^ was observed for the combination of immobilized mAb against calcitonin and labeled pAb against PCT (Fig. 4).

The concentration of antibodies used to prepare a stable immunoprobe with gold nanoparticles was 15 µg mL^−1^ for GNSs (20 and 35 nm) and GNPNs, 20 µg mL^−1^ for GNSs (50 nm), and 40 µg mL^−1^ for GNSs (70 and 100 nm) and GNSTs. Thus, nanospheres of larger size demand higher antibody concentrations during the preparation of stable conjugates and led to the consumption of specific reagents. In contrast, hierarchical 100 nm GNPNs require about half the amount of antibodies than GNSs and multi-branched GNSTs of the same size. In order to choose the optimal dilution of gold nanoparticle–antibody conjugates for LFIA, the optical density of solutions at an appropriate wavelength was varied from 0.5 to 4. Sufficient color intensity of the test line with low background was achieved by the spreading of pAb-GNS with optical density of 2, pAb-GNPN, and pAb-GNST with optical density of 4. The optimal concentration of the immobilized mAb and protein A on the test and control line, respectively, was 0.5 mg mL^−1^.

### Comparison of LFIA Based on GNSs, GNPNs, and GNSTs

**Fig. 5:**
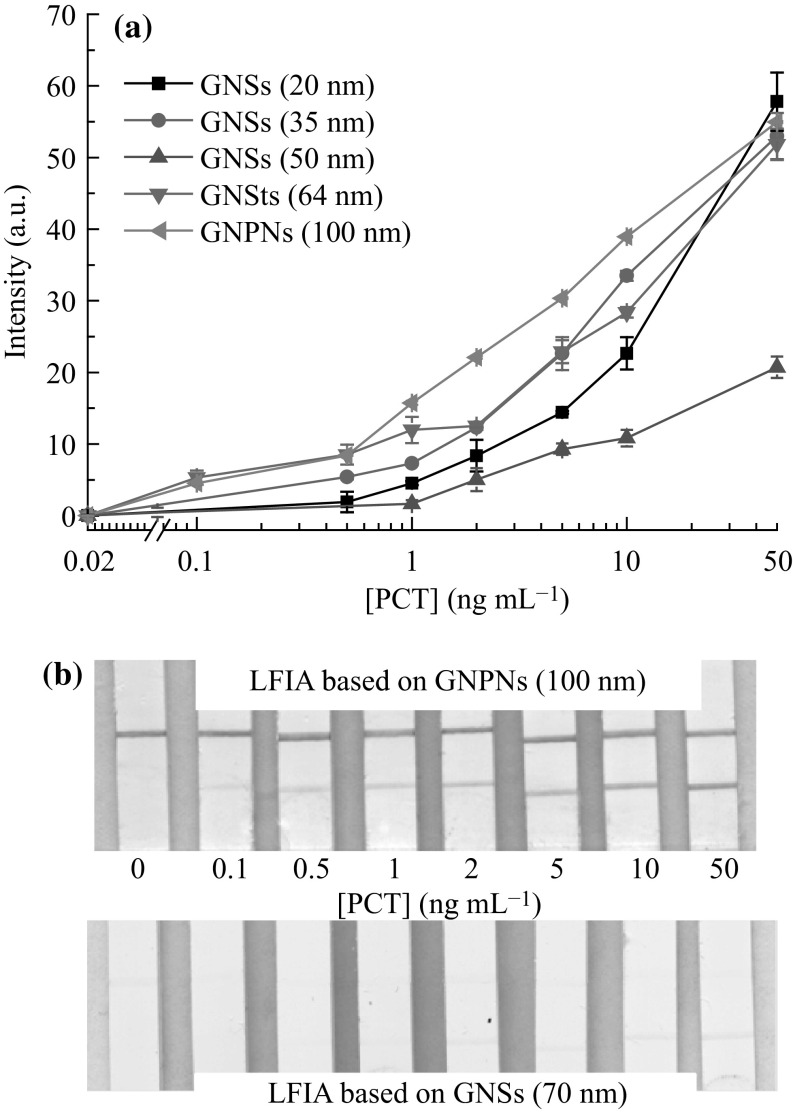
**a** Calibration curves of LFIA for PCT detection based on GNSs, GNPNs, and GNSTs. **b** Color intensity in the analytical zone for GNPN-based and GNS-based LFIA (70 nm)

According to the chosen optimal conditions, the calibration curves of LFIA based on different nanoparticles were obtained for the accurate comparison of the effects of labels on the sensitivity of analysis (Fig. 5a). It was found that the sensitivity of LFIA improved with an increase in the size of GNSs (up to 40 nm), consistent with the previous data [12]. On the other hand, the use of hierarchical large gold nanoparticles (GNPNs and GNSTs) in LFIA allowed the detection of lower PCT concentrations. Of all seven obtained gold nanoparticles, GNPN-based LFIA exhibited linearity over the range of 0.5–10 ng mL^−1^ with the best limit of detection at 0.1 ng mL^−1^ concentration, which was five times better than the sensitivity of the conventional LFIA with 20 nm GNSs. The comparison between large GNS-and GNPN-based LFIA showed that the shape, rather than the particle size, affected the sensitivity of the analysis. The sandwich complex formed after the completion of the assay was seen as a band with color change from red to gray, owing to different sizes and shapes of gold nanoparticles. The results of LFIA based on GNSs with a size of 70 and 100 nm were difficult to interpret because of the pale color of the label in the test zone of the strip (Fig. 5b). On the other hand, the hierarchical gold nanoparticles with a size around 100 nm displayed a contrasting color, which may be visually detected on the test zone of the test strip. This may be attributed to the red shift in the surface plasmon resonance peak for hierarchical-structured gold nanoparticles. For instance, 100 nm GNSs had a surface plasmon resonance peak at 582.7 nm, while 100 nm GNPNs displayed a surface plasmon resonance peak at 683 nm (Fig. 3). Multi-branched GNSTs have found wide applications in photoacoustic imaging [32], photothermal therapy [33], and biosensing [34], given their unique light scattering and absorption properties. The characteristics of GNST-based LFIA were comparable with those of conventional LFIA based on GNSs with a size of less than 50 nm. This result may be associated with the steric hindrance that occurs as a result of many tips and the uneven distribution of antibodies on the surface of GNSTs. As a consequence, the adsorption of the binding complex of antigen-labeled antibodies on the surface of GNSTs may be difficult.

**Fig. 6:**
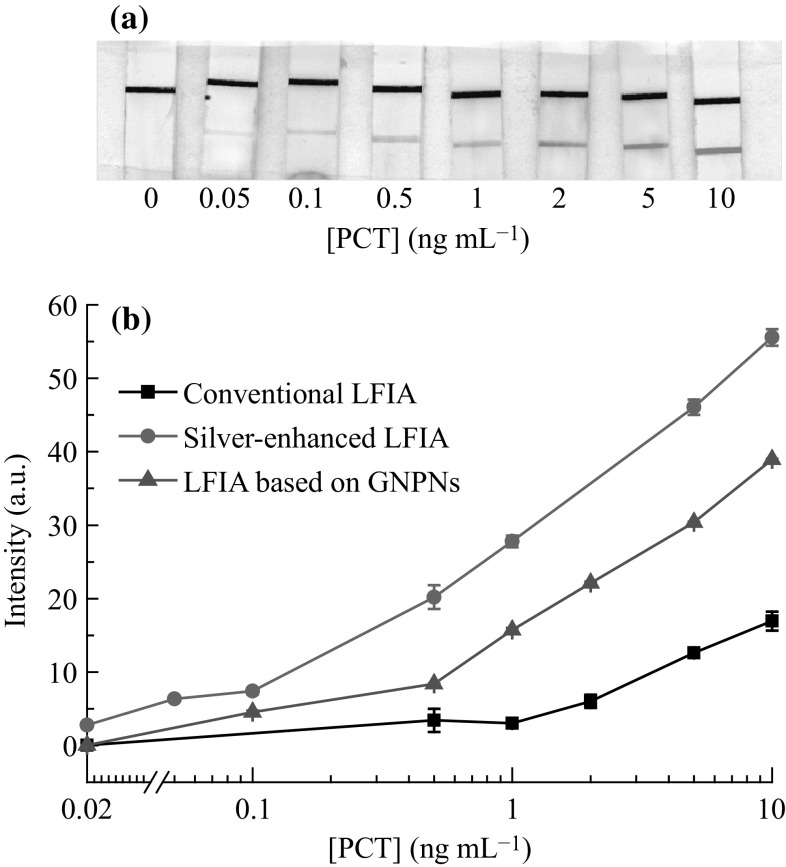
**a** Silver-enhanced LFIA for the detection of PCT. **b** Calibration curves for PCT obtained from LFIA based on 20 nm GNSs, GNPNs, and silver-enhanced LFIA

One of the effective approaches for the amplification of line intensities in the test zone of the strips includes the use of silver-enhanced labeling method. The procedure of silver-enhanced LFIA for the detection of PCT was similar to that of the conventional LFIA. After the formation of a colored red band in the test zone of the membrane associated with sandwich complex, the silver-enhancing mixture was applied. The color of the test and control zone changed from red to black. A scanned image of test strips after silver enhancement and typical calibration curves of the silver-enhanced LFIA and 20 nm GNS-based LFIA are presented in Fig. 6. The treatment of the test strip with silver-enhancing mixture resulted in the detection of PCT at 0.05 ng mL^−1^ concentration, which corresponded to a tenfold increase in the sensitivity. The results obtained are comparable with the recently reported study of silver-enhanced LFIA for prostate specific antigen [35].

Thus, the use of multi-branched GNPNs instead of GNSs as a colored label may improve the sensitivity of LFIA for PCT detection by approximately fivefold. Despite the tenfold increase in the sensitivity with the silver enhancement procedure, this approach requires additional reagents as well as operation steps of analysis and takes more time for completion as compared with the conventional GNS-based LFIA (20 vs. 10 min). In addition, the silver-enhanced method is difficult to apply outside the laboratory. For clinical purpose, the serum of patients is applied to the sample pad of LFIA test strip. The analysis is semi-quantitative, and the result is observed by the presence or absence of a colored line in the test zone of the strip. The analysis time is within 10–15 min, and the test may be performed outside the laboratory, thereby meeting the requirements of point-of-care testing. Preliminary studies have revealed the feasibility of LFIA for PCT detection in serum samples. Further studies will be focused on PCT LFIA validation and application.

## Conclusions

In this study, a new type of nanogold label was considered to improve the sensitivity of PCT LFIA. It was shown that the size and shape of the label affects the sensitivity of LFIA. To our knowledge, GNSTs and GNPNs were the first applied labels in sandwich-format LFIA. The increase in the size of GNSs (up to 40 nm) enhances the sensitivity of LFIA, but at the same time increases the antibody consumption (cost of the test). However, no increase in the antibody consumption was observed with hierarchical nanoparticles such as GNPNs. Thus, the use of GNS larger than 40 nm as a label in LFIA may reduce the color brightness and nanoparticle stability. On the contrary, hierarchical large gold nanoparticles display contrasting color, which may be easily interpreted, both visually and quantitatively. In addition, the use of GNPNs as a label demonstrated a fivefold increase in the sensitivity as compared to conventional LFIA based on 20 nm GNSs. A tenfold improvement in the sensitivity with a PCT detection limit of 0.05 ng mL^−1^ was achieved with the use of silver-enhanced LFIA. However, LFIA test based on hierarchical GNPNs contains all components in their dried forms and no additional steps and reagents are required. Thus, LFIA utilizing various complex gold nanostructures may be used to improve the sensitivity of different analytes, including the diagnostically important markers.
